# Do you cov me? Effect of coverage reduction on metagenome shotgun sequencing studies

**DOI:** 10.12688/f1000research.16804.4

**Published:** 2020-01-22

**Authors:** Federica Cattonaro, Alessandro Spadotto, Slobodanka Radovic, Fabio Marroni

**Affiliations:** 1 IGA Technology Services Srl, Udine, Udine, 33100, Italy; 2 Department of Agricultural, Food, Environmental and Animal Sciences (DI4A), University of Udine, Udine, 33100, Italy

**Keywords:** high-throughput sequencing, metagenome, metagenomics, next generation sequencing, alpha diversity, complex matrices

## Abstract

Shotgun metagenomics sequencing is a powerful tool for the characterization of complex biological matrices, enabling analysis of prokaryotic and eukaryotic organisms and viruses in a single experiment, with the possibility of reconstructing *de novo* the whole metagenome or a set of genes of interest. One of the main factors limiting the use of shotgun metagenomics on wide scale projects is the high cost associated with the approach. We set out to determine if it is possible to use shallow shotgun metagenomics to characterize complex biological matrices while reducing costs. We used a staggered mock community to estimate the optimal threshold for species detection. We measured the variation of several summary statistics simulating a decrease in sequencing depth by randomly subsampling a number of reads. The main statistics that were compared are diversity estimates, species abundance, and ability of reconstructing *de novo* the metagenome in terms of length and completeness. Our results show that diversity indices of complex prokaryotic, eukaryotic and viral communities can be accurately estimated with 500,000 reads or less, although particularly complex samples may require 1,000,000 reads. On the contrary, any task involving the reconstruction of the metagenome performed poorly, even with the largest simulated subsample (1,000,000 reads). The length of the reconstructed assembly was smaller than the length obtained with the full dataset, and the proportion of conserved genes that were identified in the meta-genome was drastically reduced compared to the full sample. Shallow shotgun metagenomics can be a useful tool to describe the structure of complex matrices, but it is not adequate to reconstruct—even partially—the metagenome.

## Introduction

Shotgun metagenomics offers the possibility to assess the complete taxonomic composition of biological matrices and to estimate the relative abundances of each species in an unbiased way ^1, 2^. It allows to agnostically characterize complex communities containing eukaryotes, bacteria and also viruses.

Metagenome shotgun high-throughput sequencing has progressively gained popularity in parallel with the advancing of next-generation sequencing (NGS) technologies ^3, 4^, which provide more data in less time at a lower cost than previous sequencing techniques. This allows the extensive application to study the most various biological mixtures such as environmental samples ^5, 6^, gut samples ^7– 9^, skin samples ^10^, clinical samples for diagnostics and surveillance purposes ^11– 14^ and food ecosystems ^15, 16^. Another, more traditional approach currently used to assign taxonomy to DNA sequences is based on the sequencing of target conserved regions. Metabarcoding method relies on conserved sequences to characterize communities of complex matrices. These include the highly variable region of 16S rRNA gene in bacteria ^17^, the nuclear ribosomal internal transcribed spacer (ITS) region for fungi ^18^ , 18S rRNA gene in eukaryotes ^19^, cytochrome c oxidase sub-unit I ( *COI* or *cox1*) for taxonomical identification of animals ^20^, *rbcL*, *matK* and *ITS2* as the plant barcode ^21^. Metabarcoding has the advantage of reducing sequencing needs, since it does not require sequencing of the full genome, but just a marker region. On the other hand, given the commonly used approaches, characterization of microbial and eukaryotic communities requires different primers and library preparations ^22^. In addition, bias in the amplification of the targeted sequence is a major issue in targeted metagenomics studies and constitutes an important limitation of metabarcoding ^23^. Several studies suggested that whole shotgun metagenome sequencing is more effective in the characterization of metagenomics samples compared to target amplicon approaches, with the additional capability of providing functional information regarding the studied approaches ^24, 25^.

Current whole shotgun metagenome experiments are performed obtaining several million reads ^5, 7^. However, obtaining a broad characterization of the relative abundance of different species might be achieved with lower number of reads.

To test this hypothesis, we analyzed ten samples (eight sequenced in the framework of this study and two retrieved from the literature) derived from different complex matrices using whole metagenomics approach and tested accuracy of several summary statistics as a function of the reduction of the number of reads used for analysis. The selection of samples belonging to different matrices with distinct characteristics enabled to understand if the results are generally applicable and, if this is not the case, which are the features with the greatest impact on results.

In summary, the aim of the present work is to test the effect of the reduction of sequencing depth on 1) estimates of diversity and species richness in complex matrices; 2) estimates of abundance of the species present in the complex matrix, and 3) completeness of *de novo* reconstruction of the genome of the species present in the samples. To assess the consistency of our approach, we selected samples characterized by different levels of species richness and by different relative abundance of prokaryotic and eukaryotic organisms and viruses.

## Methods

### Samples description and DNA extraction

The following samples were used in the present work: the mock community DNA sample “20 Strain Staggered Mix Genomic Material” ATCC ^®^ MSA-1003 ^TM^ (short name: A1), two biological medicines (B1 and B2), two horse fecal samples (F1 and F2), three food samples (M1, M2, and M3), and two human fecal samples (V1 and V2).

Biological medicines were two different lots of live attenuated MPRV vaccine, widely used for immunization against measles, mumps, rubella and chickenpox in infants. Lyophilised vaccines were resuspended in 500 μl sterile water for injection and DNA extracted from 250 μl using Maxwell ^®^ 16 Instrument and the Maxwell ^®^ 16 Tissue DNA Purification Kit (Promega, Madison, WI, USA) according to the manufacturer's instructions. The vaccine composition declared by the producer is the following: live attenuated viruses: 1) Measles (ssRNA) Swartz strain, cultured in embryo chicken cell cultures; Mumps (ssRNA) strain RIT 4385, derived from the Jeryl Linn strain, cultured in embryo chicken cell cultures; Rubella (ssRNA) Wistar RA 27/3 strain, grown in human diploid cells (MRC-5); Varicella (dsDNA) OKA strain grown in human diploid cells (MRC-5).

Horse feces from two individuals were processed as follows: 100 mg of starting material stored in 70% ethanol were used for DNA extraction using the QIAamp PowerFecal DNA Kit (QIAGEN GmbH, Hilden, Germany), according to the manufacturer's instructions.

Food samples were raw materials of animal and plant origin, used to industrially prepare bouillon cubes. DNA extractions from those three samples were performed starting from 2 grams of material each, using the DNeasy mericon Food Kit (QIAGEN GmbH, Hilden, Germany), according to the manufacturer's instructions. The declared sample composition was *Agaricus bisporus* for M1, spice ( *Piper nigrum*) for M2 and mix of animal extracts for M3.

The mock community declared components are: 0.18% Acinetobacter baumannii (ATCC 17978), 0.02% Actinomyces odontolyticus (ATCC 17982), 1.80% Bacillus cereus (ATCC 10987), 0.02% Bacteroides vulgatus (ATCC 8482), 0.02% Bifidobacterium adolescentis (ATCC 15703), 1.80% Clostridium beijerinckii (ATCC 35702), 0.18% Cutibacterium acnes (ATCC 11828), 0.02% Deinococcus radiodurans (ATCC BAA-816), 0.02% Enterococcus faecalis (ATCC 47077), 18.0% Escherichia coli (ATCC 700926), 0.18% Helicobacter pylori (ATCC 700392), 0.18% Lactobacillus gasseri (ATCC 33323), 0.18% Neisseria meningitidis (ATCC BAA-335), 18.0% Porphyromonas gingivalis (ATCC 33277), 1.80% Pseudomonas aeruginosa (ATCC 9027), 18.0% Rhodobacter sphaeroides (ATCC 17029), 1.80% Staphylococcus aureus (ATCC BAA-1556), 18.0% Staphylococcus epidermidis (ATCC 12228), 1.80% Streptococcus agalactiae (ATCC BAA-611), 18.0% Streptococcus mutans (ATCC 700610).

DNA purity and concentration were estimated using a NanoDrop Spectrophotometer (NanoDrop Technologies Inc., Wilmington, DE, USA) and Qubit 2.0 fluorimeter (Invitrogen, Carlsbad, CA, USA).

Human fecal samples V1 and V2 derive from a study investigating the virome composition of feces of uncontacted Amerindians ^26^. Data are publicly available on Sequence Read Archive (SRA, https://www.ncbi.nlm.nih.gov/sra/). The two samples with the highest sequencing depth were chosen; accession numbers are SRR6287060 and SRR6287079, respectively.

### Whole metagenome DNA library construction and sequencing

DNA library preparations were performed according to manufacturer’s protocol, using the kit Ovation ^®^ Ultralow System V4 1–96 (Nugen, San Carlos, CA). Library prep monitoring and validation were performed both by Qubit 2.0 fluorimeter (Invitrogen, Carlsbad, CA, USA) and Agilent 2100 Bioanalyzer DNA High Sensitivity Analysis kit (Agilent Technologies, Santa Clara, CA). Obtained DNA concentrations were as follows: A1 8 ng/µl (total amount = 640 ng), B1 10.7 ng/µl (total amount = 535 ng), B2 9.41 ng/µl (total amount = 470.5 ng), F1 42.3 ng/µl (total amount = 4,230 ng), F2 22.6 ng/µl (total amount = 2,260 ng), M1 16.6 ng/µl (total amount = 1,494 ng), M2 1.87 ng/µl (total amount = 168.3 ng), M3 16 ng/µl (total amount = 640 ng).

Cluster generation was then performed on Illumina cBot and flowcell HiSeq SBS V4 (250 cycle), and sequenced on HiSeq2500 Illumina sequencer producing 125bp paired-end reads.

Samples F1 and F2 were loaded on flowcell HiSeq Rapid SBS Kit v2 (500 cycles) producing 250bp paired-end reads. The estimated library insert sizes were: 539 bp (A1), 531 bp (B1), 536 bp (B2), 620 bp (F1), 620 bp (F2), 342 bp (M1), 178 bp (M2), 496 bp (M3). Samples were sequenced in different runs and pooled with other libraries of similar insert sizes.

The CASAVA Illumina Pipeline version 1.8.2 was used for base-calling and de-multiplexing. Adapters were masked using cutadapt ^27^. Masked and low quality bases were filtered using erne-filter version 1.4.6. ^28^.

### Bioinformatics analysis

**Figure 1.**
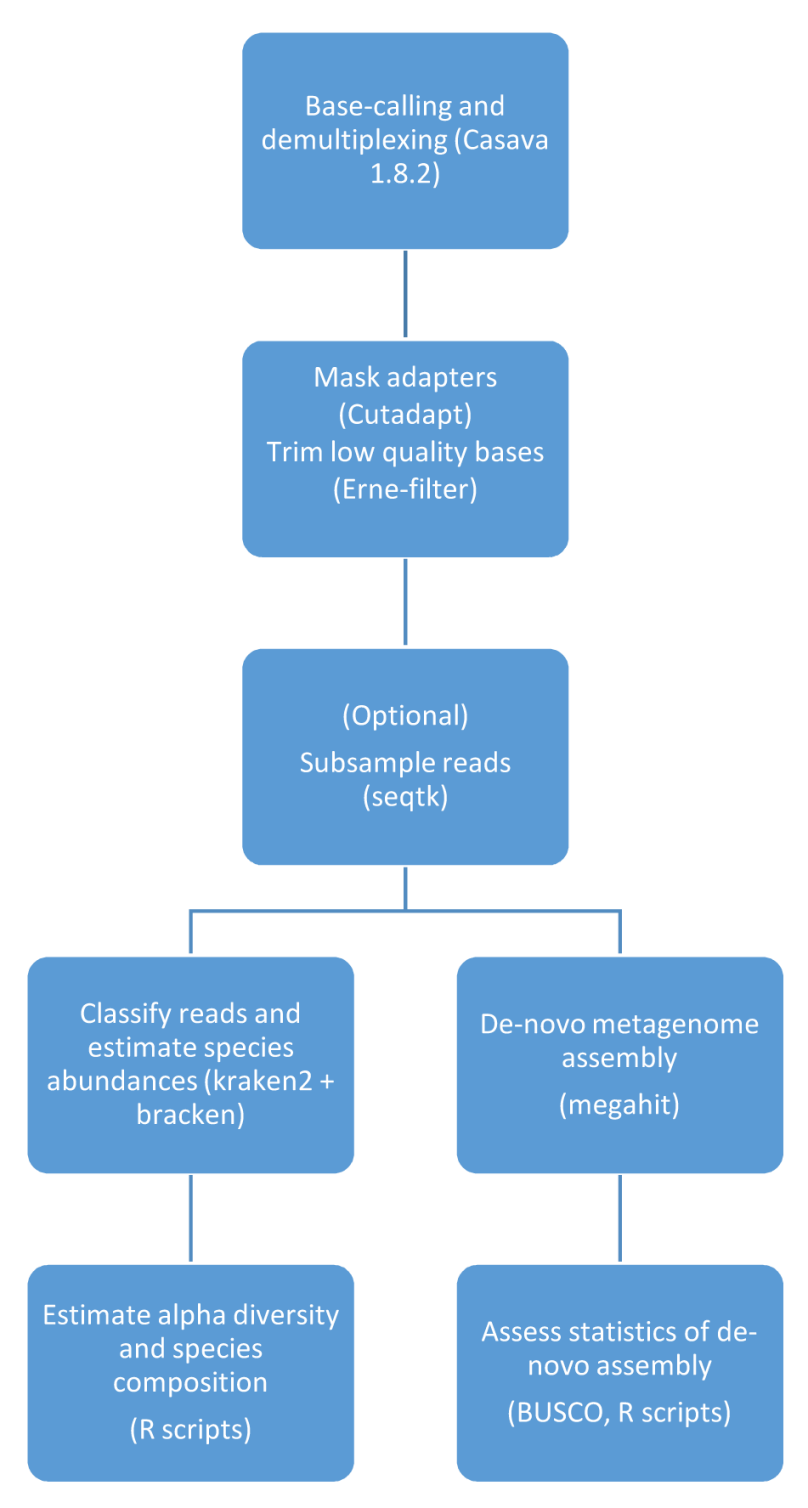
Workflow of the main bioinformatics analysis performed in the present work.

The bioinformatics analysis performed in the present work are summarized in Figure 1; a standard pipeline for reproducing the main steps of analysis is available on GitHub ( http://www.doi.org/10.5281/zenodo.2593798).

Since different read lengths among samples may constitute an additional confounder in analysis, 250 bp long reads belonging to F1, F2, V1 and V2 were trimmed to a length of 125bp using fastx-toolkit version 0.0.13 ( http://hannonlab.cshl.edu/fastx_toolkit/) before analysis.

Reduction in coverage was simulated by randomly sampling a fixed number of reads from the full set of reads. Subsamples of 10,000, 25,000, 50,000, 100,000, 250,000, 500,000 and 1,000,000 reads were extracted from the raw reads using seqtk version 1.3. To estimate the variability due to random effects, subsampling was replicated five times for each simulated depth and 99% confidence limits were estimated and plotted.

To classify the largest possible number of prokaryotes, eukaryotes and viruses, reads were classified against the complete NCBI nt database using kraken2, version 2.0.6 ^29^. The nt database was converted to kraken2 format using the built-in kraken2-build script with default parameters. Among the most significant parameters, kmer size for the database is by default set to 35 and the minimizer length to 31. A simplified representation of species composition was obtained using Krona ^30^. To obtain accurate species abundances Bracken, version 2.2 ^31^ was used on species supported by at least 10 reads; since the reads used in all the experiments were 125bp, the bracken database was built using 125bp kmers.

The threshold for declaring a species as present was set according to results of a performance analysis on the mock community (A1) for which species presence and abundance was known. Performance was assessed using F1-score, calculated as 2*TP/(2*TP+FP+FN), as previously reported ^32^. F1-score is a measure used in performance analysis when the number of true negatives is extremely high or unknown.

The effect of the selected database on reads classification was assessed on the mock community full sequencing experiment, by observing the variation in present and absent species when using different databases. In addition to the nt database built explicitly for this study, the standard kraken2 database, the minikraken2 v1 database and the minikraken2_v2 were used. The standard kraken2 database contains complete genomes in RefSeq for the bacterial, archaeal, and viral domains, along with the human genome and a collection of known vectors (UniVec_Core), and the minikraken2 v1 database contains RefSeq bacteria, archaea, and viral libraries, and the minikraken2_v2 database contains RefSeq bacteria, archaea, and viral libraries and the GRCh38 human genome.

Bracken database was built for 125bp kmers for the standard database. Minikraken2 instead is distributed as a prebuilt database, from which it is not possible to build the bracken database, but for which bracken databases with kmers distribution of 100bp, 150bp and 200 kmers are available. kmers 100bp and 150bp were tested, since they are the closest to the read length used in this study.

Observed number of taxa, Shannon’s diversity index ^33^ and Pielou’s index ^34^ were estimated using the R package vegan version 2.4.2 ^35^ or base R, version 3.3.3 ^36^ functions. The number of observed taxa was computed as the number of species passing the detection threshold.

Shannon diversity index is estimated as

$$H\;=\;–\;\sum_{i\;=\;1}^{N}p_{i}*\;\text{ln}\;(p_{i})\;$$

Where *N* is the total number of species and *p _i_* is the frequency of the species *i*.

Pielou’s evenness index is estimated as

$$J=\frac{H}{\text{In}\;S}$$

Where *H* is Shannon’s diversity index and S is the total number of observed species. The value *ln S* corresponds to the maximum possible value of *H,* observed when all species have the same frequency.

The effect of sequencing depth on beta-diversity was assessed using the procrustes and protest functions, implemented in vegan.

Assembly of the metagenome was performed using megahit version 1.1.2 ^37^ with default parameters, with kmer sizes varying as follows: 21, 29, 39, 59, 79, 99, 119, 141. Reconstructed contigs were binned at the species level using kraken2, and only contigs assigned to species above the detection threshold were used for further analysis. Completeness of the assemblies of each species was assessed using BUSCO ^38^. For each species, the proportion of the reconstructed genes was measured as the proportion of genes that were fully reconstructed, plus the proportion of genes that were partially reconstructed. For each sample, results were then averaged over detected species to provide the average proportion of reconstructed genes. BUSCO analysis was performed on prokaryotic database for all the samples with the exception of M1 (predominantly composed by fungi) for which the fungal database was used.

Unless otherwise specified, all the analysis were performed using R 3.3.3 ^36^.

## Results

### Determination of detection threshold

The mock community sample “20 Strain Staggered Mix Genomic Material” (ATCC ^®^ MSA-1003 ^TM^) was used as a reference to control performance of sequencing and classification procedures at various depth. The community includes a total of 20 bacterial species, of which 5 have a frequency of 0.02%, 5 a frequency of 0.18%, 5 a frequency of 1.8% and 5 a frequency of 18%.

Results of the performance analysis on the mock dataset are shown in Table 1. The highest F1 score (0.8) was obtained when applying a 0.1% threshold. Using this threshold, 14 species were correctly identified while 6 of them were not detected. Five out of the 6 undetected species had a nominal frequency of 0.02%; the sixth undetected species was *Helicobacter pylori*, with a nominal frequency of 0.18%, for which we recorded a frequency of 0.096%, below the 0.1% threshold. The only false positive was *Shigella flexneri*, a species highly related to *Escherichia coli* ^39^, that was observed at a frequency of 0.128%. Based on these results we used a threshold of 0.1% for declaring a species as present in a sample.

**Table 1.** Results of performance analysis. **Threshold (%)**: detection threshold, expressed as percentage of assigned reads. **TP**: true positives. **FP**: false positive. **FN**: false negatives. **F1**: F1 score.

Threshold (%)	TP	FP	FN	F1
0.001	19	188	1	0.17
0.005	19	49	1	0.43
0.01	19	32	1	0.54
0.05	15	6	5	0.73
0.1	14	1	6	0.8
0.5	10	0	10	0.67

### Sample composition

Summary statistics for the samples included in the study are shown in Table 2.

**Table 2.** Summary statistics for the full samples included in the study.

Sample	N reads	N species
A1	4,969,245	15
B1	11,031,061	4
B2	3,830,083	9
F1	12,472,553	127
F2	10,780,450	126
M1	1,898,011	5
M2	1,558,975	138
M3	1,867,879	21
V1	1,300,221	84
V2	2,001,984	12

**Sample:** Short name of the sample. **N reads:** Number of reads obtained for the sample in the full sequencing experiment. **N species**: number of species identified in the sample

**Figure 2.**
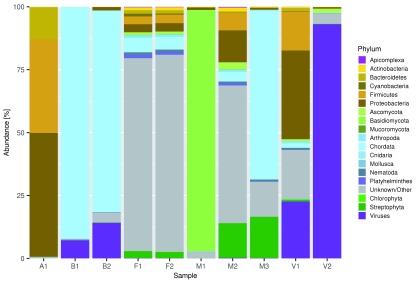
Phylum composition of the samples. Only phyla represented by at least 1% of the reads are shown. Viruses are presented at division level. Unclassified reads and reads assigned to rare phyla are aggregated under the name “Unknown/Other”.

The number of reads obtained in the samples selected for the present study ranged from slightly more than 1 million (sample V1) to more than 12 million (sample F1). The number of species identified in each sample ranged from 4 in sample B1 to 138 in sample M2. Figure 2 summarizes the composition of each sample at the Phylum level. Viruses are aggregated at the division level. Only phyla more abundant than 1% were plotted. Reads that were either unclassified or assigned to rare phyla were aggregated under the name “Unknown/Other”. Samples B1, B2 and M3 where mainly composed of Chordata, sample M1 was mostly composed of Basidiomycota, and sample V2 was mainly composed of Viruses. Samples F1, F2 and, to a lesser extent, M2 were characterized by a large proportion of reads unclassified or assigned to rare phyla. For a more detailed view of raw taxonomy composition, interactive html Chrona are available for download on Open Science Framework ( https://osf.io/y7c39/), under the project “Do you cov me”, DOI: 10.17605/OSF.IO/Y7C39.

### Effect of the choice of database

The effect of the selected database on reads classification was assessed only on the mock community. Results are shown in Table 3. All the Spearman correlation coefficients were >0.9 (not shown). Estimated abundances according to the minikraken2 v1 database were very similar to those obtained according to the minikraken2 v2 database (irrespective of the kmer length which slightly altered the results). The main differences between the two were observed for species not declared in the mock community ( *i.e.* false positives, indicated by ND in Abundance column), such as *Homo sapiens*, *Bacillus anthracis* and *Salmonella enterica*.

**Table 3.** Effect of the database choice on the assignment of species in the mock community.

Species	Abundance	v1_100	v1_150	v2_100	v2_150	standard	nt
Acinetobacter baumannii	0.18	0.219	0.219	0.219	0.219	0.177	0.326
Actinomyces odontolyticus	0.02	NP	NP	NP	NP	NP	NP
Bacillus cereus	1.8	4.002	4.039	3.962	4	3.567	4.259
Bacteroides vulgatus	0.02	NP	NP	NP	NP	NP	NP
Bifidobacterium adolescentis	0.02	NP	NP	NP	NP	NP	NP
Clostridium beijerinckii	1.8	3.027	3.023	2.975	2.971	2.524	4.69
Cutibacterium acnes	0.18	0.136	0.136	0.137	0.137	0.134	0.134
Deinococcus radiodurans	0.02	NP	NP	NP	NP	NP	NP
Enterococcus faecalis	0.02	NP	NP	NP	NP	NP	NP
Escherichia coli	18	23.793	23.986	23.619	23.95	25.945	20.043
Helicobacter pylori	0.18	NP	NP	NP	NP	NP	NP
Lactobacillus gasseri	0.18	NP	NP	NP	NP	NP	0.103
Neisseria meningitidis	0.18	0.118	0.118	0.118	0.119	0.121	0.12
Porphyromonas gingivalis	18	11.834	11.829	11.844	11.84	11.811	11.706
Pseudomonas aeruginosa	1.8	3.067	3.078	3.055	3.065	3.145	3.233
Rhodobacter sphaeroides	18	22.588	22.568	22.448	22.426	23.22	23.047
Staphylococcus aureus	1.8	2.098	1.994	1.781	1.734	2.494	1.537
Staphylococcus epidermidis	18	13.76	13.942	13.821	13.938	13.112	16.701
Streptococcus agalactiae	1.8	1.067	1.075	1.079	1.089	0.645	1.026
Streptococcus mutans	18	10.898	10.889	10.877	10.864	10.851	11.543
Bacillus anthracis	ND	NP	NP	0.125	0.114	NP	NP
Bacillus thuringiensis	ND	0.359	0.343	0.322	0.311	0.773	NP
Escherichia albertii	ND	0.194	0.194	0.179	0.179	NP	NP
Escherichia marmotae	ND	0.113	0.113	0.111	0.111	NP	NP
Homo sapiens	ND	NP	NP	0.109	0.11	0.116	NP
Salmonella enterica	ND	0.97	0.697	1.573	1.165	NP	NP
Shigella dysenteriae	ND	0.239	0.242	0.209	0.212	NP	NP
Shigella flexneri	ND	NP	NP	NP	NP	NP	0.128
Staphylococcus lugdunensis	ND	NP	NP	NP	NP	0.108	NP
Streptococcus pyogenes	ND	NP	NP	NP	NP	0.495	NP

**Species:** Binomial nomenclature of the species. **Abundance:** declared abundance. **v1_100:** estimated abundance using minikraken2 v1 database and bracken database kmer length of 100. **v1_150:** estimated abundance using minikraken2 v1 database and bracken database kmer length of 150. **V2_100:** estimated abundance using minikraken2 v2 database and bracken database kmer length of 100. **V2_150:** estimated abundance using minikraken2 v2 database and bracken database kmer length of 150. **standard**: estimated abundance of species using standard database. **nt**: estimated abundance of species using nt database. **ND**: Not declared in the mock community. **NP**: Not present according to the detection threshold of 0.1%.

*Homo sapiens* contamination was detected with the Minikraken2 v2 (the only Minikraken2 containing human sequences) and using the standard database. No contamination was detected using the nt database, for which *Homo sapiens* was recorded with a frequency of 0.086% and was therefore below the detection threshold of 0.1%. *Bacillus anthracis* was only detected when using the Minikraken2 v2 database. *Salmonella enterica* was detected in all the experiments involving the Minikraken2 databases, but it was the only species for which substantial variation in the estimated frequencies was observed, ranging from 0.697% to 1.573% according to Minikraken2 v1 with 150 kmers and Minikraken2 V2 with 100 kmers, respectively.

False positives were generally low: the total contribution of false positives ranged from 0.168% for nt to 2.628% for Minikraken2 v2 100 kmers. However, none of the databases was immune to false positives. The nt database showed only one false positive, *Shigella flexneri,* while minikraken and standard databases showed more than one false positive each.

The estimated abundances of the declared species were in excellent agreement across databases, with some exceptions. For *Escherichia coli*, with declared abundance of 18%, estimated abundances ranged from 20.043% to 25.954% when using nt and the standard database, respectively. *Staphylococcus epidermidis*, with declared abundance of 18%, was estimated at 13.112% by the standard database and 16.701% by nt. Finally, *Clostridium beijerinckii*, declared at 1.8% was estimated at 2.524% by the standard Kraken databse and at 4.69% by nt database.

### Species abundance

**Figure 3.**
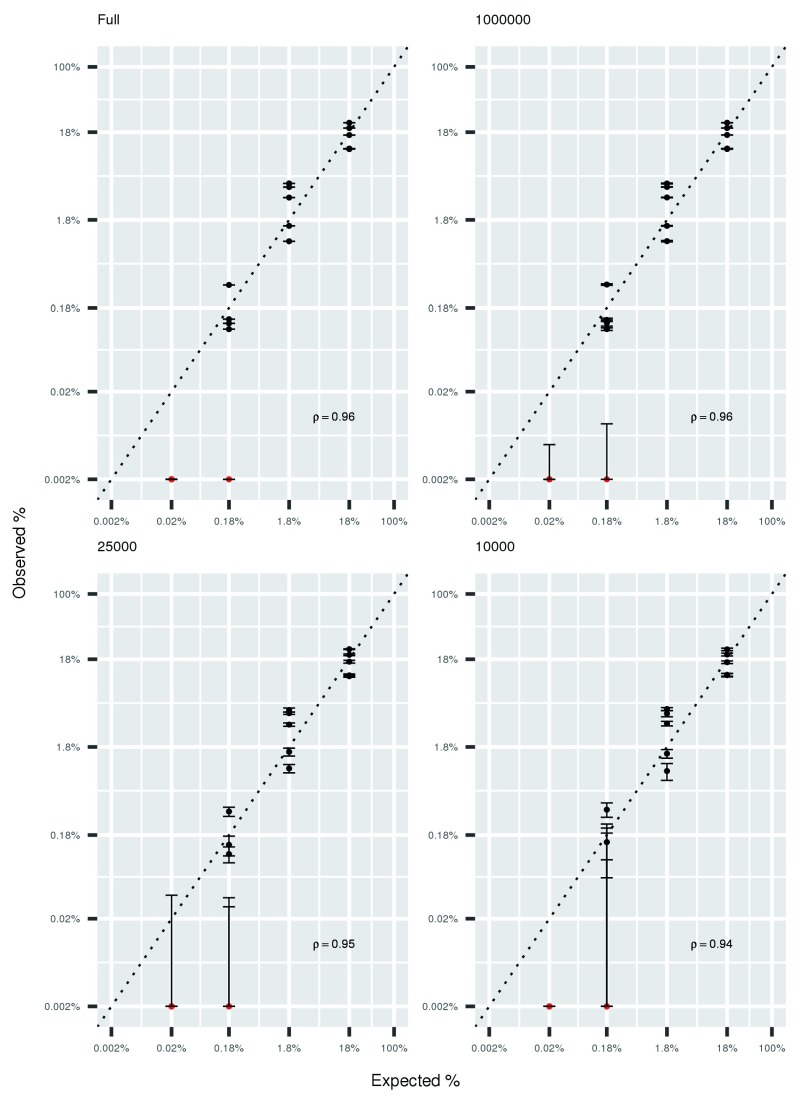
Observed and expected abundance of bacterial species present in the mock community “20 Strain Staggered Mix Genomic Material” (ATCC ^®^ MSA-1003 ^TM^) at varying sequencing depths. In red, species identified at frequency lower than the selected threshold of 0.1% and arbitrarily plotted at 0.002%. Error bars represent 95% confidence intervals obtained from five resampling experiments. Both axes are plotted in log scale to facilitate visualization of rare species.

The effect of reducing sequencing depth on the accuracy of taxonomical classification was assessed by using the mock community, given its known composition. Expected and observed abundancies of the 20 mock species maintained a high correlation (r=0.94) even when decreasing sequencing to 10,000 reads ( Figure 3). However, decreasing sequencing depth caused an increase in uncertainty, as shown by the broader confidence intervals for lower depths, particularly for rare species.

**Figure 4.**
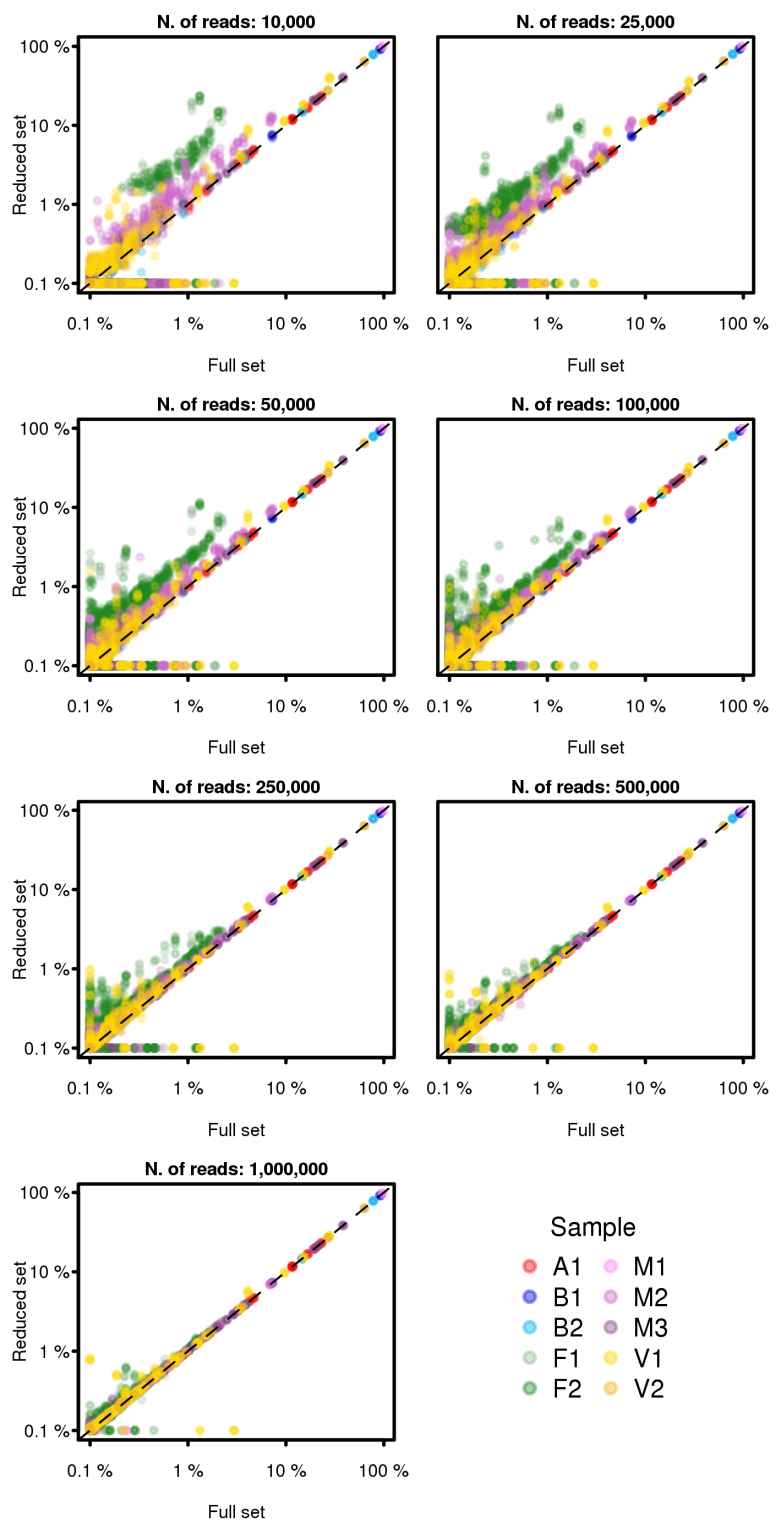
Correlation of species abundance estimated using full and reduced datasets. Data for all the five subsampled replicates are plotted. Each point (colored by sample of origin) represents a given species. Both axes are plotted in log scale to facilitate visualization of rare species.

We also measured the correlation in species abundance between the full and reduced datasets in all the samples ( Figure 4). The correlation between the two quantities was in general high and improved at increasing sequencing depths. The average Spearman’s correlation coefficient between full and reduced samples ranged from 0.71 in the 25,000 reads subsample to 0.94 in the 1,000,000 reads subsample.

### Diversity analysis

**Figure 5.**
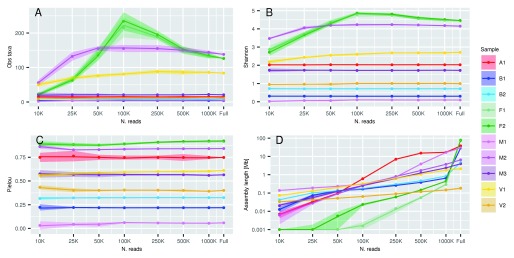
Effect of reduction of sequencing depth on: **A**) Observed number of taxa, **B**) Shannon’s diversity index, **C**) Pielou’s diversity index, and **D**) Total length of *de novo* assembly. In all panels X axis is in log scale and Y axis is in linear scale with the exception of panel F, in which both axes are in log scale. Shaded areas represent the confidence limits of resampling experiments. The number of reads used for the analysis is shown from the smallest (10,000) on the left, to the full dataset on the right.

We further evaluated the impact of reducing sequencing depth on several diversity measures, such as the observed number of taxa, Shannon’s diversity index and Pielou’s diversity index ( Figure 5).

Samples F1, F2, M2 and V1 had more than 50 taxa, and all the remaining samples had less than 25 ( Table 2 and Figure 5A). Downsampling only produced significant differences in the four samples with high number of observed taxa (panel A). In samples F1 and F2, intermediate levels of downsampling ( *e.g.* 100,000 reads) caused an increase in the number of species exceeding the 0.1% abundance threshold.

Shannon’s diversity index (panel B) is a widely used method to assess biological diversity of ecological and microbiological communities. The effect of sequencing depth on Shannon’s diversity index is negligible for most samples, with the exception of the samples with the richest species composition (F1, F2, and M2) for which downsampling led to a significant variation in the estimate.

Pielou’s index (panel C) is a measure of the species’ distribution evenness. Values close to 1 denote equifrequent species, and lower values denote uneven distribution of species relative abundance. The effect of the number of reads on Pielou’s index is negligible for all samples.

**Figure 6.**
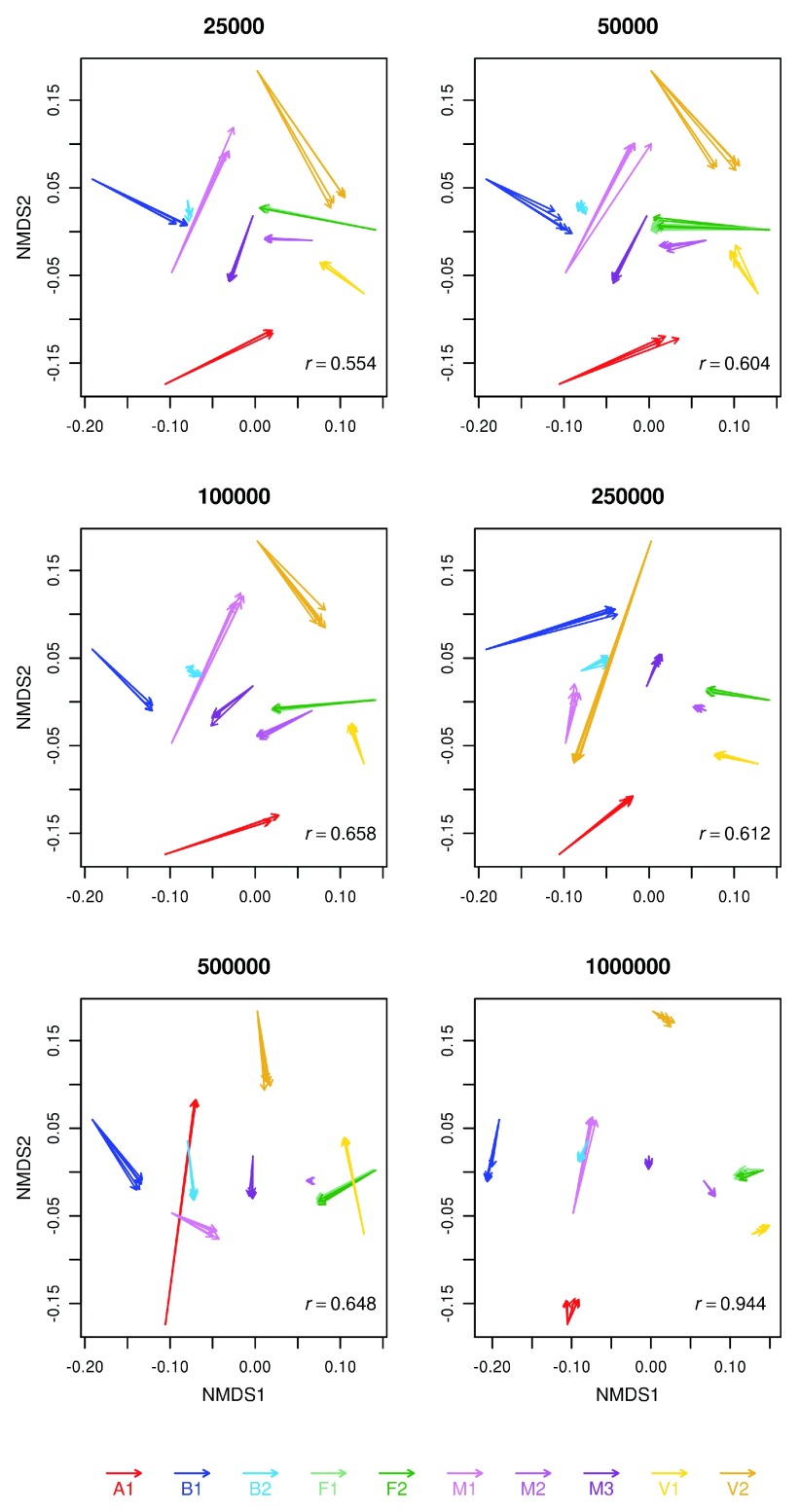
Procrustes analysis. Arrows start from the NMDS matrix of the full sample and the arrowhead ends at the NMDS matrices of the five subsampled replicates for each sample. The correlation between the matrices are shown at the bottom right of each plot. All correlations were significant (p<=0.001).

Effect of sequencing depth on beta-diversity using procrustes analysis is shown in Figure 6. Procrustes analysis is shown between the full dataset (starting point of the arrows) and each replicate of each of the reduced dataset (arrival points of the arrowheads). The smallest dataset was excluded for the analysis because it did not have sufficient information for procrustes analysis. The relative positions between the full dataset and the reduced datasets tended to remain similar across different subsampling, with some notable exception, such as V2 in the 250,000 reads sample and A1 in the 500,000 sample. F1 and F2, always clustered together. Correlation was good between all the matrices, but it was excellent (r>0.9) only for the comparison of the full dataset with the 1,000,000 reads subsample.

### Completeness of *de novo* assembly

We investigated the effect of coverage reduction on the completeness of *de novo* assembly. We reconstructed the metagenome of the full and reduced datasets and compared the completeness of the reconstructed genomes. Results are summarized in Figure 5 (panel D). As expected, the size of the assembly was strongly influenced by the sequencing depth. Assembly size for the full dataset ranged from less than 1 Mb (V2) to nearly 100 Mb (F1 and F2). A decrease in the sequencing depth led to a steady decrease in assembly size in all samples. At 1,000,000 reads the size ranged from slightly more than 100 kb (V2) to slightly more than 10Mb (A1 and M1).

BUSCO analysis ^38^ was used as an additional measure to assess the completeness of the reconstructed metagenome.

**Figure 7.**
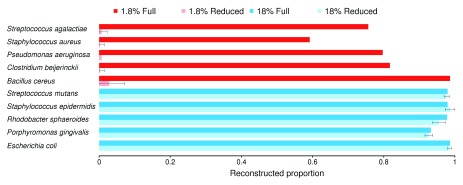
Proportion of BUSCO genes reconstructed in the full set of reads, and in a subset of 1,000,000 reads. Error bars represent 95% confidence intervals based on five subsampling experiments. **1.8%:** Species with nominal abundance of 1.8%; **18%**: Species with nominal abundance of 18%; **Full:** results using full set of reads; **Reduced;** results using a subsample of 1,000,000 reads.

First, we assessed the performance on the A1 mock community for the full set of reads (4,969,245 reads) and for the largest subset, (1,000,000 reads). Genomes of species present at 0.02% and 0.18% were not reconstructed, while genomes of species present at 1.8% or 18% were reconstructed. The proportion of BUSCO genes reconstructed using the whole set of reads ranged 59%–99% for species present at 1.8% and 93%–99% in the most abundant species ( Figure 7). At 1,000,000 reads the proportion of reconstructed BUSCO genes dropped to 0.7%–3% for species present at 1.8% while it ranged 93%–99% for the species present at 18%.

**Figure 8.**
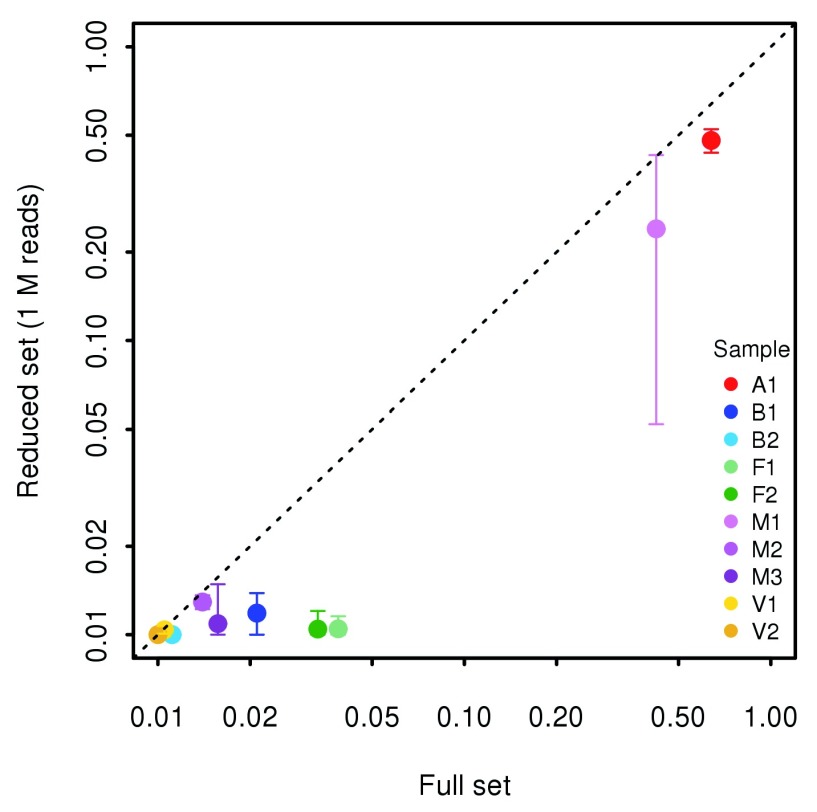
Completeness of the BUSCO genes in the full dataset (X axis) and in the largest of the reduced datasets (consisting of 1,000,000 reads, Y axis); error bars are based on the five replicate experiments performed for each sample. The plot is in log-log scale.

In addition, we plot in Figure 8 the proportion of reconstructed genes in full (X axis) and reduced (Y axis) datasets obtained by randomly sampling 1,000,000 reads. The proportion of reconstructed BUSCO genes is very low even in the full samples, indicating that in general the sequencing depth is still too low to obtain an accurate reconstruction of the metagenome. Only in samples A1 and M1, the average proportion of BUSCO genes reconstructed in the full sample was greater than 10%. Reducing sequencing depth to 1,000,000 reads significantly lowered the proportion of reconstructed genes in all the samples, as testified by the fact that all the points and their confidence limits lie below the diagonal.

## Discussion

We set out to test the effect of the reduction of sequencing depth in metagenome shotgun sequencing experiments on 1) estimates of diversity and species richness; 2) estimates of species abundance, and 3) completeness of *de novo* reconstruction of the genome of the species present in complex matrices. We selected ten heterogeneous samples that underwent whole genome DNA-sequencing. This was also true for vaccine samples B1 and B2, several components of which are ssRNA viruses, and could not be detected using this approach. Indeed, the determination of the ssRNA components in vaccines was not the aim of the present study.

We used the mock community to determine the optimal detection threshold and then performed all the analysis enforcing the selected threshold. Five of the species composing the mock community had a declared abundance of 0.02% and could not by definition be detected using the threshold. However, the threshold caused the appearance of only one false positive, and resulted in a F1 score of 0.8. The false positive species is *Shigella flexneri* a sister species of *Escherichia coli*, and is likely a result of misclassification of a proportion of reads. A possible explanation is that in the used database, genomic sequence of *Escherichia coli* is classified as *Shigella flexneri*. We thus further investigated if the use of different databases could change this behavior, by classifying the mock community reads against the standard database and the two Minikraken2 databases distributed with kraken2. We noticed that while the performance was overall in excellent agreement, there were some differences in the results obtained with each database. In particular, each database recorded at least one false positive species. Thus, we suggest researchers to cautiously interpret results, especially when unexpected species are identified.

To the best of our knowledge, this is the first published work reporting the observed frequencies of a mock community using shotgun high-throughput sequencing. However, previous studies performed very extensive analysis on target 16s sequencing of mock communities, and reported large deviations from the expected values, dependent on sequencing primers, extraction method and sequencing platform ^40^. We tested the effect of decrease in sequencing depth on deviations from expected frequency ( Figure 3) and observed that even when sampling 10,000 reads the average correlation between expected and observed abundances remained high (r=0.94), although the variance among resampling experiments was high.

Horse fecal samples F1 and F2 and the food sample M2 are characterized by a large number of observed species (127, 126 and 138, respectively), while all the other samples have lower number of species, ranging from 4 in B1 to 84 in V1. The greater diversity of F1, F2 and M2 compared to others is confirmed by Shannon’s and Pielou’s indices, although Pielou’s index also assigns high variability to the mock community A1. The effect of sequencing depth on nearly all indices is moderate, although researchers should be aware that very complex samples (such as F1, F2 and M2 in our study) require high sequencing depth (1 million reads) to ensure that all indices are correctly estimated, since we observed the reduction in coverage could result in under- or over-estimation of the number of taxa and of Shannon’s diversity index ( Figures 5A and 5B).

We then set out to assess the changes in the estimated relative frequency of each individual species when reducing the number of sequenced reads. Accurate estimate of the relative abundance of each species is an important task when the aim is a) to detect species with a relative abundance above any given threshold, b) to differentiate two samples based on different abundance of any given species composition, or c) to cluster samples based on their species composition.

Our results show that species abundances can be reliably estimated for most samples even in case of substantial reduction of sequencing depth. However, researchers should be aware that for complex samples (horse fecal samples F1 and F2, in our study), extreme reduction in coverage might result in biases in the estimation of species abundances ( Figure 4).

Finally, we assessed the effect of a reduction in the sequencing coverage on the ability of reconstructing *de novo* the metagenome. Our results suggest that 1 million reads are clearly suboptimal for *de novo* assembly for all the tested samples. Assembly size obtained subsampling 1 million reads are significantly smaller than those obtained with the full depth in all samples, included M1, M2 and M3, for which the full sequencing depth was less than 2 million reads ( Figure 5D).

Additional analysis were performed to assess the effect of downsampling on the completeness of the *de novo* assembly. First, we used BUSCO to assess the completeness of assemblies of the species used in the mock community A1 sample, and to compare the performance in the full set and in the larger reduced set (1 million reads). No BUSCO genes were reconstructed for species with frequencies of 0.02% and 0.18%, and we show results only for the 10 species with frequency of 1.8% or greater. The full sequencing depth (~5 million reads) enabled the reconstruction of the majority of BUSCO genes in all the species, ranging 59% ( *Staphylococcus aureus*) to 99% ( *Bacillus cereus* and most of the species with 18% frequency). The ability of reconstructing BUSCO genes in assemblies obtained with 1 million reads was unchanged for the species with 18% abundance, while it dramatically decreased for species at frequency 1.8% ( Figure 7).

We then performed a similar analysis on all the samples. Our results show that downsampling had a strongly negative effect on the proportion of reconstructed genes in all the study samples ( Figure 8).

Our results clearly indicate that the proportion of genes reconstructed with BUSCO in the full dataset is very low for all samples, with the exception of the two samples M1, predominantly composed by one fungal species, and A1, composed by a limited number of small genomes, some of which with uniform and high abundance. In addition, detailed analysis of BUSCO performance in sample A1 revealed that only the genomes of the most frequent species could be reconstructed ( Figure 7), even at full sequencing depth, amounting to nearly 5 million reads. Reduction of sequencing depth resulted in significant reduction of performance in all samples, as shown by the fact that the point estimates of the proportion of reconstructed genes and their confidence limits are below the diagonal in Figure 8. These results indicate that a complete reconstruction of the metagenome of a complex matrix requires at least several million reads. Our conclusions are also important for research aimed at the reconstruction of an interesting part of the meta-genome, such as genes involved in antibiotic resistance ^41^. The decrease in performance observed in the genes’ reconstruction will be likely observed for any gene category. Researchers aiming at a *de novo* reconstruction of the metagenome (although partial) must keep in mind that several millions of reads are needed to attain reliable results.

Researchers should be cautious when the fraction of reads that can be used to classify the microbial community is low. This might happen if the sample includes a substantial proportion of poorly characterized organisms, *i.e.* organisms not present in current databases, or if the samples come from biopsy or blood, thus containing a large proportion of the host tissue. In both cases, the amount of reads that can be used for the classification is already much lower than the number of produced reads, and further reduction is discouraged.

In the present work we tested the feasibility of using metagenome shotgun shallow high-throughput sequencing to analyze complex samples for the presence of eukaryotes, prokaryotes and virus nucleic acids for monitoring, surveillance, quality control and traceability purposes. We show that, if the aim of the experiment is a taxonomical characterization of the sample or the identification and quantification of species, a low-coverage shotgun high-throughput sequencing is a good choice, provided that at least 500,000 reads are sequenced. On the other hand, if one of the aims of the study relies on *de novo* assembly, substantial sequencing efforts are required. The number of reads required for the reconstruction of the meta-genome, depends on several factors such as the number of species in the sample, their genome size and abundance and length of the sequencing reads. An estimation needs to be performed for each experiment based on specific goals and sample characteristics.

## Data availability

### Underlying data

Raw reads generated in the present study are available at NCBI Sequence Read Archive.

Sample A1 is available under accession number SRP174028: https://identifiers.org/insdc.sra/SRP174028.

Samples F1 and F2 are available under accession number SRP163102: https://identifiers.org/insdc.sra/SRP163102.

Samples B1 and B2 are available under accession number SRP163096: https://identifiers.org/insdc.sra/SRP163096;

and samples M1, M2 and M3 are available under accession number SRP163007: https://identifiers.org/insdc.sra/SRP163007.

### Extended data

Open Science Framework: Do you cov me. https://doi.org/10.17605/OSF.IO/Y7C39 ^42^.

This project contains the raw html graphs, produced using Krona.

## Software availability

Pipeline for performing the standard analysis included in this work available from: https://github.com/fabiomarroni/doyoucovme.

Archived code at time of publication: https://doi.org/10.5281/zenodo.2593798 ^27^.

License: GNU GPL-3.0.
