# Supplementary material for: Do you cov me? Effect of coverage reduction on metagenome shotgun sequencing studies
Source: F1000Res. 2020 Jan 22;7:1767. Originally published 2018 Nov 8. [Version 4] doi: 10.12688/f1000research.16804.4 (PMC7059852; doi:10.12688/f1000research.16804.4)
Supplement: Peer review history

# Peer review: Reviewer response for version 4

**DOI:** 10.5256/f1000research.24347.r58937

**Published:** 2020-03-04

**Authors:** Francesco Dal Grande

**Affiliations:** 1 Senckenberg Biodiversity and Climate Research Centre, Senckenberg Gesellschaft für Naturforschung, Frankfurt am Main, Germany; 2 LOEWE Centre for Translational Biodiversity Genomics (TBG), Frankfurt am Main, Germany

After reading the authors' response, I agree that the authors have adequately addressed all the referees' comments.

I confirm that I have read this submission and believe that I have an appropriate level of expertise to confirm that it is of an acceptable scientific standard.

# Peer review: Reviewer response for version 4

**DOI:** 10.5256/f1000research.24347.r58938

**Published:** 2020-02-05

**Authors:** Marcus Claesson, Shriram Patel

**Affiliations:** 1 APC Microbiome Ireland, University College Cork, Cork, Ireland

We have now reviewed the authors’ response and agree that they have sufficiently addressed our comments.

We confirm that we have read this submission and believe that we have an appropriate level of expertise to confirm that it is of an acceptable scientific standard.

# Peer review: Reviewer response for version 3

**DOI:** 10.5256/f1000research.22041.r55903

**Published:** 2019-12-02

**Authors:** Marcus Claesson, Shriram Patel

**Affiliations:** 1 APC Microbiome Ireland, University College Cork, Cork, Ireland

This is an interesting piece of work looking to evaluate influence of varying sequencing coverage (depth) on the ability to harness information about taxonomic composition and species diversity and possible use of shallow whole metagenome shotgun sequencing as a potential cost-effective alternative to targeted 16S rRNA gene sequencing in large scale studies.

 In general, the manuscript is very well written and make use of staggered mock community along with the actual samples sequenced from diverse environmental origin to optimize required metagenomic sequencing depth to address potential research question.

 The authors have used statistics such as alpha diversity, species abundance and completeness of reconstructed genomes to evaluate performance of reduce sequencing efforts. It would be interesting, although not required, to see how overall between sample beta diversity (bray-curtis) changes with varying sequencing depth and in full datasets (considering only actual samples). This could offer insights into whether samples coming from diverse environment clusters together even at varying sequencing depth (as low as 10K)? or does reduce sequencing depth influences overall metagenome composition. Particularly, it would be interesting to see Procrustes analysis between full datasets and reduced datasets (may be at 100K/ 500K reads because estimated alpha diversity reached plateau and most of the species gets covered).

 I am confused with statement on page 8. “Intermediate level of down sampling (here 100K reads) caused an increase in observed species, due to increased number of species exceeding the 0.1% abundance cut-off (selected based on mock community)”. Does this indicate that with increased sequencing effort (particularly in horse fecal samples) those species exceeding the cut-off at reduced sequencing depth did not detected?

 It would be good if authors can add important limitation of shallow shotgun metagenomic sequencing in discussion. Particularly note on “poorly characterized samples” for which no representative genomes are available in database or “samples coming from biopsy or blood” where host DNA accounts for most of the extracted DNA.

 Some General comments:

1. All samples were trimmed to the read length of 125bp. Did authors build bracken database with default read length of 100 or 125? If so, please mention that in the manuscript.
2. Please move formula of alpha diversity indexes in method’s section.
3. In the abstract, ‘diversity’ should be prepended with ‘alpha’ as it might otherwise include beta-diversity which wasn’t analysed.
4. The title is quite long (just a matter of taste)
5. 5 ^th^ sentence in Intro: technically, fungi are also eukaryotic, so this needs to be reflected.
6. The colour scheme in Figure 2 could be improved. Currently the phyla are ordered alphabetically which is a wasted opportunity for more information. At the least, they should be ordered by kingdom. Unknown could be black/grey/white
7. Correlations for Fig 4 are Pearson, which only should be used if the data follows a normal distribution, otherwise Spearman.
8. Insert “,” for each 1,000 in N. of reads to improve readability
9. All fonts in Figure 5 are too small and unreadable

We confirm that we have read this submission and believe that we have an appropriate level of expertise to confirm that it is of an acceptable scientific standard, however we have significant reservations, as outlined above.

# Response

**Published:** 2020-01-15

**Authors:** Federica Cattonaro

**Affiliations:** IGA Technology Services Srl, Italy

**Reviewer 4**

**Marcus Claesson**, APC Microbiome Ireland, University College Cork, Cork, Ireland 

**Shriram Patel**, APC Microbiome Ireland, University College Cork, Cork, Ireland 

 This is an interesting piece of work looking to evaluate influence of varying sequencing coverage (depth) on the ability to harness information about taxonomic composition and species diversity and possible use of shallow whole metagenome shotgun sequencing as a potential cost-effective alternative to targeted 16S rRNA gene sequencing in large scale studies.

 In general, the manuscript is very well written and make use of staggered mock community along with the actual samples sequenced from diverse environmental origin to optimize required metagenomic sequencing depth to address potential research question.

 The authors have used statistics such as alpha diversity, species abundance and completeness of reconstructed genomes to evaluate performance of reduce sequencing efforts. It would be interesting, although not required, to see how overall between sample beta diversity (bray-curtis) changes with varying sequencing depth and in full datasets (considering only actual samples). This could offer insights into whether samples coming from diverse environment clusters together even at varying sequencing depth (as low as 10K)? or does reduce sequencing depth influences overall metagenome composition. Particularly, it would be interesting to see Procrustes analysis between full datasets and reduced datasets (may be at 100K/ 500K reads because estimated alpha diversity reached plateau and most of the species gets covered).

***We now performed Procrustes analysis between the full dataset and all the reduced sets (we then removed the 10K dataset, because diagnostic measures showed that the MDS on that matrix was not reliable). The analysis is now shown as Figure 6, described, and discussed.***

 I am confused with statement on page 8. “Intermediate level of down sampling (here 100K reads) caused an increase in observed species, due to increased number of species exceeding the 0.1% abundance cut-off (selected based on mock community)”. Does this indicate that with increased sequencing effort (particularly in horse fecal samples) those species exceeding the cut-off at reduced sequencing depth did not detected?

***Yes.***

***The species exceeding the cut-off at reduced sequencing depth were still “detected” at full sequencing depth, but they didn’t exceed the threshold. For example, in the fecal sample 1 (F1), In the full-depth sample, we assigned reads to 6273 species (with an average frequency of 0.02%), but only 124 of them exceeded the threshold; in the 100000 sample we assigned reads to 350 species (with an average frequency of 0.6%), 215 of which exceeded the threshold.***

***This phenomenon was observed only for the fecal samples, which are the ones with greater complexity and higher number of reads in the full sample. We rewrote part of the results to try to clearly convey our take-home message, i.e.: although reduction in coverage depth usually does not affect estimation of sample diversity, it can in some cases result in an under- or over-estimation of such quantities.***

 It would be good if authors can add important limitation of shallow shotgun metagenomic sequencing in discussion. Particularly note on “poorly characterized samples” for which no representative genomes are available in database or “samples coming from biopsy or blood” where host DNA accounts for most of the extracted DNA.

***We added the following sentence in the discussion: Researchers should be cautious when the fraction of reads that can be used to classify the microbial community is low. This might happen if the sample includes a substantial proportion of poorly characterized organisms, i.e. organisms not present in current databases, or if the samples come from biopsy or blood, thus containing a large proportion of the host tissue. In both cases, the amount of reads that can be used for the classification is already much lower than the number of produced reads, and further reduction is discouraged.***

 Some General comments:

1. All samples were trimmed to the read length of 125bp. Did authors build bracken database with default read length of 100 or 125? If so, please mention that in the manuscript.

***We built a bracken database for 125 kmers. On request of Reviewer 3 we also performed tests on different databases, only for the mock community. One of the additional databases (minikraken) comes as a prebuilt database without possibility of building the bracken index, and we used distributed databases built with 100kmers and 150kmers. We added this information in the methods section.***

  

1. Please move formula of alpha diversity indexes in method’s section.

***Done***

  

1. In the abstract, ‘diversity’ should be prepended with ‘alpha’ as it might otherwise include beta-diversity which wasn’t analysed.

***We left this unchanged, since we are now also analyzing beta-diversity, and the generic statement of the abstract is true for beta diversity as well.***

  

1. The title is quite long (just a matter of taste)

***We changed the title to: Do you cov me? Effect of coverage reduction on metagenome shotgun sequencing studies***

  

1. 5 ^th^ sentence in Intro: technically, fungi are also eukaryotic, so this needs to be reflected.

***We removed the word fungi***

1. The colour scheme in Figure 2 could be improved. Currently the phyla are ordered alphabetically which is a wasted opportunity for more information. At the least, they should be ordered by kingdom. Unknown could be black/grey/white

***We changed the colour scheme for Figure 2. Protozoan (only apicomplexan detected) are red-violet, bacteria are in shades of brown, fungi are in shades of olive green, vertebrates are in shades of blue, plants in shade of green, unknown are grey, and viruses are violet***


* *

1. Correlations for Fig 4 are Pearson, which only should be used if the data follows a normal distribution, otherwise Spearman.

***We computed correlations for data of figure 4 as Spearman. We now also present correlation of Figure 3 as spearman’s rho, for the same reason (none of the two data followed a normal distribution).***

  

1. Insert “,” for each 1,000 in N. of reads to improve readability

***Done***

  

1. All fonts in Figure 5 are too small and unreadable

***We increased the font size***

# Peer review: Reviewer response for version 3

**DOI:** 10.5256/f1000research.22041.r51765

**Published:** 2019-08-08

**Authors:** Francesco Dal Grande

**Affiliations:** 1 Senckenberg Biodiversity and Climate Research Centre, Senckenberg Gesellschaft für Naturforschung, Frankfurt am Main, Germany; 2 LOEWE Centre for Translational Biodiversity Genomics (TBG), Frankfurt am Main, Germany

I appreciate the changes made in this revision. Specifically, I am glad to see that the authors used the results for the mock community to set the parameters for detecting the presence of species in the other samples. Improved is also the inference of the relative abundances of species using bracken and the presentation of the BUSCO results.

 I have only minor suggestions that I hope will help further improving the manuscript. 

 My only issue is the detection of the false positive ( *Shigella flexneri*) for the mock community data set. I agree with the authors that this might likely be the result of misclassification of a small portion of reads. This, however, may also be the result of incorrect taxonomic profiles that may be present in the chosen (full NCBI nt) database. The evaluation of the effects of database taxonomic correctness and composition on species assignment accuracy is clearly not the scope of the present work. However, since the correct profiling of the mock community is crucial for selecting the best detection threshold for all other data sets, I suggest to strengthen the analysis of the mock community by comparing kracken/bracken results using different databases (only for the mock community): **full NCBI nt **vs. **full bacterial RefSeq** vs. **curated genome database** (i.e. including only the 20 genomes of the species forming the mock community).

 Minor points:

- In the abstract, add a line to describe the use of the mock community in your study.
- Figure 1: I would modify the box 'Classify reads (kraken2)' into 'Classify reads and estimate species abundances (kraken2 + bracken)'.
- p. 8: "The effect of the number of reads on Pielou's index is moderate". Please define 'moderate'. 
- p. 10: Please move the formulas of the two indices to the Materials and Methods section.

Other corrections:

- p. 6: M1 was mostly composed OF.
- p. 11: "..the performance in the full set and IN".
- p. 12: ".., depends on several factors such as THE number of species ..".

I confirm that I have read this submission and believe that I have an appropriate level of expertise to confirm that it is of an acceptable scientific standard, however I have significant reservations, as outlined above.

# Response

**Published:** 2020-01-15

**Authors:** Federica Cattonaro

**Affiliations:** IGA Technology Services Srl, Italy

**Reviewer 3**

**Francesco Dal Grande**, Senckenberg Biodiversity and Climate Research Centre, Senckenberg Gesellschaft für Naturforschung, Frankfurt am Main, Germany;  LOEWE Centre for Translational Biodiversity Genomics (TBG), Frankfurt am Main, Germany

 I appreciate the changes made in this revision. Specifically, I am glad to see that the authors used the results for the mock community to set the parameters for detecting the presence of species in the other samples. Improved is also the inference of the relative abundances of species using bracken and the presentation of the BUSCO results.

 I have only minor suggestions that I hope will help further improving the manuscript. 

 My only issue is the detection of the false positive ( *Shigella flexneri*) for the mock community data set. I agree with the authors that this might likely be the result of misclassification of a small portion of reads. This, however, may also be the result of incorrect taxonomic profiles that may be present in the chosen (full NCBI nt) database. The evaluation of the effects of database taxonomic correctness and composition on species assignment accuracy is clearly not the scope of the present work. However, since the correct profiling of the mock community is crucial for selecting the best detection threshold for all other data sets, I suggest to strengthen the analysis of the mock community by comparing kracken/bracken results using different databases (only for the mock community): **full NCBI nt **vs. **full bacterial RefSeq** vs. **curated genome database** (i.e. including only the 20 genomes of the species forming the mock community).

***This is a very good point. We were already aware that the choice of the database would affect the accuracy of the results, and the choice to use nt database was motivated by the fact that when studying heterogeneous samples potentially including Eukaryotes the nt would be the database of choice. We avoided by purpose to tackle the aspect of accuracy of databases taxonomic correctness. However, we agree that a simple comparison based on the mock community data would benefit the manuscript and the readers. Thus we tested the following additional databases 1) the “standard” database distributed with kraken2 which is a full bacterial+viral+fungi RefSeq database with the addition of the human genome, and 2) Several “minikraken2” databases that are distributed with kraken2 (the details on the composition of the minikraken2 are provided in the manuscript). We didn’t use the curated database only including the 20 genomes of the species forming the mock community because in that case by definition we will not identify any false positive; even in the case of a real contamination of the mock community all the classified reads would be attributed to one of the 20 genomes, because those are the only genomes present in the database.***

***Our results show a general good agreement across databases, but some differences were observed. This is especially true for the false positives; each database returns different false positives. It is possible that different databases have – minor – different classification issues. This however should motivate researchers to cautiously interpret results, especially before claiming contaminations form unexpected species in a given sample. This results are now shown in a Table and discussed.***

 Minor points:

- In the abstract, add a line to describe the use of the mock community in your study.
- ***Done ***

 

- Figure 1: I would modify the box 'Classify reads (kraken2)' into 'Classify reads and estimate species abundances (kraken2 + bracken)'.
- ***Done***

 

- p. 8: "The effect of the number of reads on Pielou's index is moderate". Please define 'moderate'. 
- ***Very good point. We changed moderate to negligible; indeed Pielou’s index is the most stable across sequencing depths.***

 

- p. 10: Please move the formulas of the two indices to the Materials and Methods section.
- ***Done***

Other corrections:

- p. 6: M1 was mostly composed OF.
- ***Done***
- p. 11: "..the performance in the full set and IN".
- ***Done***
- p. 12: ".., depends on several factors such as THE number of species ..".
- ***Done***

 

# Peer review: Reviewer response for version 2

**DOI:** 10.5256/f1000research.20298.r48341

**Published:** 2019-05-30

**Authors:** Francesco Dal Grande

**Affiliations:** 1 Senckenberg Biodiversity and Climate Research Centre, Senckenberg Gesellschaft für Naturforschung, Frankfurt am Main, Germany; 2 LOEWE Centre for Translational Biodiversity Genomics (TBG), Frankfurt am Main, Germany

In this manuscript the authors aimed at evaluating the use of shallow shotgun metagenomic sequencing for the characterisation of species diversity and the reconstruction of genomes in complex Illumina read sets. Overall, the manuscript is well written and contains interesting information that may be useful to others in figuring out a required metagenomic sequencing depth for a given goal.  

 The manuscript has been vastly improved in the current version, however I feel that it still needs a thorough revision to address a few major issues in order to ensure the general validity of the findings.

 The three major issues to address are, in my opinion, the following:

1. **Overestimation of diversity**: Authors decided to base their analyses of diversity on the raw output from kraken2. However, as mentioned by the authors themselves, "species represented by only one read are unlikely to be real". This is quite evident in the report from the 20-species mock community comprising instead >2000 species. I strongly recommend the use of a threshold (e.g., 0.005% of the total amount of reads) to filter out likely false positives. For this purpose, the authors could take advantage of the mock community to evaluate results based on different thresholds and thereby optimise threshold selection. 
2. **Inaccuracy of species-level abundances**: in their analysis the authors assumed that read abundances reflect species abundance. However, this is often not the case, especially when closely related taxa are present in the sample; the accuracy of abundance estimation further depends on the database used (Lu *et al* 2017). The authors themselves hint at this when discussing the misclassification of *Staphylococcus lugdunensis,* likely due to the presence of other confounding *Staphylococcus* reads. To address this issue, the authors could use Bracken (from the same developers of kraken, Lu *et al. *2017). Bracken uses the classification results of kraken to reestimate relative species abundances taking into account how much sequence from each species is identical to other genomes in the database.
3. **Inaccurate assessment of genome reconstruction ability**: considering the classification biases mentioned above and the complexity of the investigated metagenomic data sets, it might be better to base the assessment of the effects of coverage reduction on metagenome reconstruction solely on the mock community data. First, authors would need to bin the metagenomic contigs into individual species (using kraken2 and/or other binning approaches). The individual bins (i.e., species) should then be evaluated for completeness using BUSCO and  compared.

In summary, this work (and, by extension, future studies using a similar approach) could greatly benefit from the inclusion of a baseline estimate for species diversity and metagenome reconstruction, even if it is derived from a single mock community. The additional data sets could then be used to validate these estimates against real data.

I confirm that I have read this submission and believe that I have an appropriate level of expertise to state that I do not consider it to be of an acceptable scientific standard, for reasons outlined above.

## References

[1] : Bracken: estimating species abundance in metagenomics data. *PeerJ Computer Science*.2017;3: 10.7717/peerj-cs.104 10.7717/peerj-cs.104 PMC1201628240271438

# Response

**Published:** 2019-07-23

**Authors:** Federica Cattonaro

**Affiliations:** IGA Technology Services Srl, Italy

***In this manuscript the authors aimed at evaluating the use of shallow shotgun metagenomic sequencing for the characterisation of species diversity and the reconstruction of genomes in complex Illumina read sets. Overall, the manuscript is well written and contains interesting information that may be useful to others in figuring out a required metagenomic sequencing depth for a given goal.***

***The manuscript has been vastly improved in the current version, however I feel that it still needs a thorough revision to address a few major issues in order to ensure the general validity of the findings.***

We thank the reviewer for the suggestions. We implemented them and updated the manuscript accordingly.

***The three major issues to address are, in my opinion, the following: ***

1. ***Overestimation of diversity: Authors decided to base their analyses of diversity on the raw output from kraken2. However, as mentioned by the authors themselves, "species represented by only one read are unlikely to be real". This is quite evident in the report from the 20-species mock community comprising instead >2000 species. I strongly recommend the use of a threshold (e.g., 0.005% of the total amount of reads) to filter out likely false positives. For this purpose, the authors could take advantage of the mock community to evaluate results based on different thresholds and thereby optimise threshold selection.  ***
  See answer to point 2.
2. ***Inaccuracy of species-level abundances: in their analysis the authors assumed that read abundances reflect species abundance. However, this is often not the case, especially when closely related taxa are present in the sample; the accuracy of abundance estimation further depends on the database used (Lu et al 2017). The authors themselves hint at this when discussing the misclassification of Staphylococcus lugdunensis, likely due to the presence of other confounding Staphylococcus reads. To address this issue, the authors could use Bracken (from the same developers of kraken, Lu et al. 2017). Bracken uses the classification results of kraken to reestimate relative species abundances taking into account how much sequence from each species is identical to other genomes in the database.***
  We took advantage of suggestions 1 and 2 (and from suggestions from reviewer 1) to improve the species abundances estimation. After classifying reads with kraken2, we used bracken to re-estimate species abundance only for species represented by at least 10 reads. Then, using the only gold standard we had (the mock community) we measured performance at difference detection threshold. Our results suggested that a detection threshold of 0.1% was the one resulting in the higher F1 score, minimizing false negatives and false positives while maximizing true positives.
3. ***Inaccurate assessment of genome reconstruction ability: considering the classification biases mentioned above and the complexity of the investigated metagenomic data sets, it might be better to base the assessment of the effects of coverage reduction on metagenome reconstruction solely on the mock community data. First, authors would need to bin the metagenomic contigs into individual species (using kraken2 and/or other binning approaches). The individual bins (i.e., species) should then be evaluated for completeness using BUSCO and compared.***
  Results presented in version 2 of our paper are already based on binning approaches, in which we classified contigs using kraken, performed BUSCO for each species and then averaged the proportion of BUSCO genes across species. However, in version 2 we made (in our opinion) a mistake, since we averaged the proportion of BUSCO genes across all species for which at least one BUSCO gene was reconstructed. This led to a slight overestimation of the number of reconstructed BUSCO genes. We thus repeated the analysis by averaging the proportion of BUSCO genes over all the species that were above the detection threshold, including those for which no BUSCO gene was reconstructed. The new approach is now explained in the methods section, and the new plot is now Figure 7. In addition, we liked the idea of using the mock community, and we performed a new analysis, now shown in Figure 6. The result are very interesting and are briefly discussed. Basically, with the full set of reads (around 5M), the majority of BUSCO genes could be reconstructed for species with a nominal abundance of 18% and 1.8%, but not for the rarer species (for which basically no gene could be reconstructed).  When only 1M reads are used for the assembly, the proportion of reconstructed BUSCO genes is nearly unchanged in abundant species and drops to less than 10% in species with a nominal frequency of 1.8%. The results and the implications for study designs are briefly discussed in the paper.

# Peer review: Reviewer response for version 2

**DOI:** 10.5256/f1000research.20298.r46099

**Published:** 2019-03-25

**Authors:** José F. Cobo Diaz

**Affiliations:** 1 Laboratoire Universitaire de Biodiversité et Ecologie Microbienne, IBSAM, ESIAB, Université de Brest, Plouzané, France

I appreciate the changes make along the introduction, because the objective of the present study is now more clear. Although the manuscript was improved considerably, there is still a big problem with the data analysis, mainly in reads filtering.

 Now that you have included a mock community sample, you need to use this sample to adapt the parameters of reads filtering, clustering step (I asume you have done some kind of clustering since you talk about singletons) and taxonomic assignation until you have the number of species expected, 20 in this case. You can also have some less due to problems with species assignation, but it is crazy to use a 20 species mock community and say that you have 2571 species in this sample. For example, singletons (clustering groups or OTUs (Operational Taxonomical Units) with a unique sequence) are usually removed on metabarcoding pipelines, and in some cases OTUs with less than 0.1% of abundance are removed, assuming that these sequences are sequencing errors (and PCR errors in metabarcoding). Therefore, you have to estimate the minimum percentage of abundance to be considered real (and not due to errors) with the mock sample and apply this cut off value to the rest of samples.

 In the same line, to say that 2,507 and 4,597 species were found in vaccines is not correct, where you can expect the DNA from varicella (the other viruses are ssRNA) and the DNA from human and chicken cells used for culture.

 Some small changes I suggest:

- Rewrite or suppress last paragraph of introduction, which looks more appropriate to Methodology.
- Add some disadvantages of use metabarcoding approach (being the main one the bias due to primers, with over/under-estimation of some taxa, depending of the primers used).
- At the end of the samples description, you need to put what means SRA (and add the corresponding web-address).
- In samples description, grammatical mistake with human faecal (have to be human fecal).
- Remove this sentence from results: To ensure that our conclusions have a general validity, we selected samples originating from very different sources with different compositions, and sequenced them at different depths.
- Figure 3, with species and genus level is enough.

Thus, the read filtering and hence all the statistical analysis have to be re-make. I not expect big changes, also at taxonomical level (where only a reduction of "rare species" and unclassified sequences is expected), but it is not convenient to present the results with such great over-estimation of species richness.

I confirm that I have read this submission and believe that I have an appropriate level of expertise to state that I do not consider it to be of an acceptable scientific standard, for reasons outlined above.

# Response

**Published:** 2019-07-23

**Authors:** Federica Cattonaro

**Affiliations:** IGA Technology Services Srl, Italy

***I appreciate the changes make along the introduction, because the objective of the present study is now more clear. Although the manuscript was improved considerably, there is still a big problem with the data analysis, mainly in reads filtering.***

***Now that you have included a mock community sample, you need to use this sample to adapt the parameters of reads filtering, clustering step (I asume you have done some kind of clustering since you talk about singletons) and taxonomic assignation until you have the number of species expected, 20 in this case. You can also have some less due to problems with species assignation, but it is crazy to use a 20 species mock community and say that you have 2571 species in this sample. For example, singletons (clustering groups or OTUs (Operational Taxonomical Units) with a unique sequence) are usually removed on metabarcoding pipelines, and in some cases OTUs with less than 0.1% of abundance are removed, assuming that these sequences are sequencing errors (and PCR errors in metabarcoding). Therefore, you have to estimate the minimum percentage of abundance to be considered real (and not due to errors) with the mock sample and apply this cut off value to the rest of samples.***

***In the same line, to say that 2,507 and 4,597 species were found in vaccines is not correct, where you can expect the DNA from varicella (the other viruses are ssRNA) and the DNA from human and chicken cells used for culture.***

According to your suggestions (and to similar suggestions received from reviewer 3), we now adopted more stringent criteria for determining the presence of a species. Following the suggestion of both reviewers, we leverage the mock community to define a threshold. We use Bracken to refine the species abundance estimation (already providing a very permissive threshold, i.e. ignoring OTUs with less than 10 reads). We then performed a performance analysis to compare Bracken results with the known composition of the mock community, and chose the threshold maximizing the F1 score (harmonic average of precision and recall). The threshold resulting in the best tradeoff was 0.1%.  

As a side effect of filtering OTUs with less than 0.1% frequency we do not have any narrow-sense singleton. As a consequence, the number of observed taxa and Chao1 diversity index coincide, and the Good estimator is always 1. We thus removed these two statistics from our panel plot.

In addition, we removed the paragraph on the “detection threshold” and the corresponding Table 2, since we are now determining a threshold *a-priori* based on the mock community and this parts are not needed any more.

***Some small changes I suggest: ***

- ***Rewrite or suppress last paragraph of introduction, which looks more appropriate to Methodology. ***

We removed the last paragraph.

- ***Add some disadvantages of use metabarcoding approach (being the main one the bias due to primers, with over/under-estimation of some taxa, depending of the primers used). ***

We added a sentence and a reference regarding limitation of metabarcoding approaches in the introduction.

- ***At the end of the samples description, you need to put what means SRA (and add the corresponding web-address). ***

Done.

- ***In samples description, grammatical mistake with human faecal (have to be human fecal). ***

Amended.

- ***Remove this sentence from results: To ensure that our conclusions have a general validity, we selected samples originating from very different sources with different compositions, and sequenced them at different depths. ***

Sentence removed.

- ***Figure 3, with species and genus level is enough. ***

While we were modifying the Figure as per reviewer’s request we realized that indeed the results presented at the species level in Figure 3 are also presented in the first panel of Figure 4. Since the results at the genus species did not add much information, we decided to remove Figure 3.

# Peer review: Reviewer response for version 1

**DOI:** 10.5256/f1000research.18370.r42422

**Published:** 2019-01-04

**Authors:** José F. Cobo Diaz

**Affiliations:** 1 Laboratoire Universitaire de Biodiversité et Ecologie Microbienne, IBSAM, ESIAB, Université de Brest, Plouzané, France

The authors proposed and evaluated the influence of reduce sequencing effort (amount of sequences) for a whole metagenome shotgun analysis, using the Illumina platform, in the species composition and diversity index of the communities studied. Although the idea and hypothesis are good, some problems were found in the experimental design and data analysis.

 According to the questions proposed in the peer review form, it is not a new method, only the adaptation of a current methodology to optimize the cost and increase the potential numbers of samples analyzed per run of Illumina platform. Although the introduction is clearly explained, the reasons for use shotgun sequencing, mainly to analyze viruses data and functional data for all the organism, no emphasis on such points was done in the results and discussion. The samples used (vaccines, horse fecal samples and food samples) and the introduction remark the detection of pathogens as the main objective of the approach used, including viruses, which can not be screened by amplicons approaches, like metabarcoding sequencing. I suggest adapting the text and manuscript to focus on pathogens (mainly viruses) found along the sub-samples taken for each sample. At that point, some contaminated samples (or not contaminated samples mixed with known amounts DNA from pathogen viruses) have to be used to determine the lowest pathogen concentration that could be detected for each shotgun sequencing coverage proposed.

 Many problems were found with the methodology employed, mainly the parameters used in each step and/or software employed for data filtering and analysis, which are critical for the results, which can have strong variations depending of the parameters used. Hence, the methodology proposed does not allow any replication of the method used. Moreover, there are some mistakes for species designation in the study, with at least 2508 species found in vaccine samples indicating big problems along read filtering and data analysis, because this number of species is often found in more complex systems, such as soils samples from agricultural fields. Moreover, go to species classification using some taxonomical markers, such ITS or 16SrRNA, is risky with sequences lower than 400 bp, and sometimes with bigger sequences. In the current manuscript, the use of non taxonomical marker sequences and 150 bp lengths increase enormously the number of sequences not correctly assigned to species level, and in several cases also for higher taxonomical levels (genus, family...). Therefore, I suggest to clarify how the species assignment was done, because it looks like that each gene-species was considered as one species, and each gene found for a single species was counted as a new species.

 Alpha diversity indexes employed are not the best ones, in my opinion, to describe or compare the sub-samples proposed in this manuscript. The chao1 index, an estimator of richness, has a strong influence on the number of singletons obtained in the samples, which due to the complexity of the samples-data tends to be high. Shannon index is influenced by both richness (number of taxa) and evenness (equability, Pielou index), and the reduction of richness due to the loss of rare taxa has a strong influence on this index. I propose to use the number of observed taxa instead of estimated taxa, and any evenness index, like the Pielou index, instead of the Shannon index. Moreover, the use of a coverage index, such Good’s coverage index, could be useful to compare the loss of information associated to sampled size or coverage.

 In conclusion, although the raw data can contains some important information, the manuscript has to be improved with new “pathogen contaminated” samples, and be re-written to focus on the detection of pathogens in the samples, which due to the low abundance of the samples could not be detected depending of the shotgun coverage.

I confirm that I have read this submission and believe that I have an appropriate level of expertise to state that I do not consider it to be of an acceptable scientific standard, for reasons outlined above.

# Peer review: Reviewer response for version 1

**DOI:** 10.5256/f1000research.18370.r40445

**Published:** 2018-11-27

**Authors:** Alejandro Sanchez-Flores

**Affiliations:** 1 Institute of Biotechnology, National Autonomous University of Mexico (UNAM)), Cuernavaca, Mexico

The authors propose and evaluate a whole metagenome shotgun analysis via a low sequencing yield approach, using the Illumina platform.

 In general, the idea and hypothesis are good, but the experimental design itself lacks important controls and there are many variables that are not analyzed and that can potentially bias the results.

 My main concern is that the used samples have many variables and despite using a "replicate" for each case, samples within the same type were very different. Also the nature of each sample could have an effect in the DNA isolation, in particular for the vaccine ones. Also, regarding the vaccines, it is not clear to me, if what they are looking for is DNA of potential contaminants, since all viruses in the vaccine are ssRNA. That would be my guess, but is not clear from the text.

 The main problem is that to test the influence of the sequencing yield, it would be extremely important to know the initial DNA concentration of each organism in the sample. Therefore, a mock metagenome or controlled sample would be much better as a reference to compare real life cases. In real life cases, the presence of certain organisms detected by the presence of its DNA, is not necessarily an indicator of the availability of alive organisms. Depending on the case, the presence of just the organism DNA could be an indicator of contamination which in the case of vaccines could be really bad. However, in the case of food material, finding DNA of pathogens, has to be associated with microbiology tests. However, with low sequencing yield, is very probable that very DNA in low amounts will be missed, even if this is not changing diversity indexes such as Chao1 and Shannon.

 Finally, the main difference where low yield has a significant impact can be observed in the fecal samples. This is expected since among all the tested samples, fecal ones are the most diverse and sub-sampling will really affect them as observed in Figure 3.

 Since the composition of each sample is not known *a priori*, then there are some factors that can contribute to biases. As mentioned, the DNA concentration but also its integrity (fragmentation) will affect the library construction; the cited kit requires DNA amplification which will have a bias towards GC rich genomic regions; library size was not described and was not mentioned if the samples were pooled with other libraries with different insert sizes, which affect not only the sequencing quality but the yield.

 In terms of bioinformatics analysis, it will be required to put the parameters used for each program, in case someone wants to reproduce this. For Kraken2, it is important to know what is the kmer size to index the database. For MEGAHIT assembly it will be important to know the kmer and step sizes used. For the completeness assessment, the authors used BUSCO, but apparently they are using the whole assembly to assess the completeness. This is not correct, since they must first separate in bins which genomes they have really reconstructed and then they can assess the completeness of them. Probably they can report the an average completeness value for all the reconstructed genomes. By doing the binning they can have a better analysis of what was really reconstructed and how complete it was.

 The use of Krona in Figure 2 is not very convenient. The whole point of a Krona graph is that is interactive. If authors want to provide the Krona data to be downloaded it would be possible and recommended. Having said that, I recommend to use bar plots to represent the relative abundance and composition of the samples at a given taxa level.

 Again, the idea is very good but the work needs to be improved before indexing.

I confirm that I have read this submission and believe that I have an appropriate level of expertise to state that I do not consider it to be of an acceptable scientific standard, for reasons outlined above.

# Response

**Published:** 2018-11-30

**Authors:** Federica Cattonaro

**Affiliations:** IGA Technology Services Srl, Italy

We are grateful for the constructive comments. We agree with all of them and we are planning corrective actions, listed below.

***My main concern is that the used samples have many variables and despite using a "replicate" for each case, samples within the same type were very different.***

The observation is correct. Actually, the diversity of the samples was sought by purpose in order to be able to generalize the conclusions of our paper. The fact that diversity estimate and species abundance estimation remain reliable even with strong down-sampling for all of the samples is encouraging us to think that this is a general (although not necessarily universal) observation. The same is true for the observation that de-novo assembly quickly loses accuracy when decreasing the number of sequenced reads. Maybe this wasn’t made clear enough in the paper, and we will clarify it.

***Also the nature of each sample could have an effect in the DNA isolation, in particular for the vaccine ones.***

Quantities of DNA isolated from vaccine samples (B1 and B2) were estimated to be ~2 µg using Qbit fluorimeter. However, we will provide a table with all the details about quantity, concentration, quality and size of starting DNA for all samples used in the study.

***Also, regarding the vaccines, it is not clear to me, if what they are looking for is DNA of potential contaminants, since all viruses in the vaccine are ssRNA. That would be my guess, but is not clear from the text.***

The vaccine composition declared by the producer is the following:

Live attenuated viruses: Measles (ssRNA) Swartz strain, cultured in embryo chicken cell cultures; Mumps (ssRNA) strain RIT 4385, derived from the Jeryl Linn strain, cultured in embryo chicken cell cultures; Rubella (ssRNA) Wistar RA 27/3 strain, grown in human diploid cells (MRC-5); Varicella (dsDNA) OKA strain grown in human diploid cells (MRC-5).

By DNA-seq we expected to find Varicella (dsDNA) OKA strain DNA (which was found and confirmed by variant analysis with respect to AB097932.1 Human herpesvirus 3 DNA, sub strain vOka). In addition, we found also human and chicken DNA. For human’s, we confirmed MRC-5 cell origin by mitochondrial genome variant analysis.

Genotyping analyses gave us confidence on the validity of the obtained results, even though they were beyond the scope of this work.

To identify vaccine’s ssRNA viruses we extracted RNA and performed RNA-seq from the same B1 and B2 samples. This aspect also goes beyond the scope of this work.

***The main problem is that to test the influence of the sequencing yield, it would be extremely important to know the initial DNA concentration of each organism in the sample. Therefore, a mock metagenome or controlled sample would be much better as a reference to compare real life cases.***

A mock community experiment is already on-going by using ‘10 Strain Staggered Mix Genomic Material (ATCC® MSA-1001™)’. Of course, the data obtained will be integrated in the analysis results.

***In real life cases, the presence of certain organisms detected by the presence of its DNA, is not necessarily an indicator of the availability of alive organisms. Depending on the case, the presence of just the organism DNA could be an indicator of contamination which in the case of vaccines could be really bad. However, in the case of food material, finding DNA of pathogens, has to be associated with microbiology tests.***

We agree with the observation of the reviewer. However, the aim of this work is to determine if low-pass whole genome sequencing can be an appropriate approach to broadly describe a complex matrix; finding and confirming contaminants in vaccines or DNA pathogens in food samples was beyond of the scope of the paper.

***However, with low sequencing yield, is very probable that very DNA in low amounts will be missed, even if this is not changing diversity indexes such as Chao1 and Shannon. Finally, the main difference where low yield has a significant impact can be observed in the fecal samples. This is expected since among all the tested samples, fecal ones are the most diverse and sub-sampling will really affect them as observed in Figure 3.***

We agree with the reviewer; we add some thoughts just to clarify. We indeed observed that extremely rare species (with frequencies lower than 1/10000) are lost when subsampling to the most extreme levels. When subsampling to 100K reads we are losing species with a frequency around 1/100,000 (very approximate estimate). However, the effect of losing such species on the global sample diversity as estimated by Shannon diversity index is negligible (see Figure 4, in which we show that reduction in sequencing depth has no dramatic effect on Shannon’s diversity index). The situation is different for the Chao 1 estimator. This is expected and is due to the way Chao1 is computed: this estimator relies heavily on the number of singletons (i.e. species represented by only one read). By subsampling, singletons (i.e. the rarest species) are very likely to be lost. The same phenomenon can be inferred by looking at Figures 5 and 6. Those represent a scatterplot of the relative abundance of species in full sample and reduce samples (100K and 10k reads, respectively). The plots are shown in log log scale to emphasize differences for low-frequency species. Only low-frequency species have some variation in frequency estimation. However, even when sampling only 10K read, species with frequency around 0.1% (i.e. 1/1000) are appropriately quantified. All of these observations led us to conclude that coverage reduction doesn’t prevent a satisfactory characterization of complex matrices (with the only exception of Chao 1 estimator).

***Since the composition of each sample is not known a priori, then there are some factors that can contribute to biases. As mentioned, the DNA concentration but also its integrity (fragmentation) will affect the library construction; the cited kit requires DNA amplification which will have a bias towards GC rich genomic regions; library size was not described.***

The Nugen Ovation® Ultralow System V4 kit used is a standard kit for NGS library preparation ( https://www.nugen.com/sites/default/files/DS_v2-Ovation_Ultralow_V2.pdf

It is a standard protocol widely used by the scientific community to perform DNA-seq also from low input DNA quantities (1 ng), even if in our case input DNA was of moderate quantity. Mock community experiment will shed light on eventual biases.

DNA concentration and integrity as well as input DNA quantities used in library construction and libraries insert size will be reported in the version 2 of the paper.

***It was not mentioned if the samples were pooled with other libraries with different insert sizes, which affect not only the sequencing quality but the yield.***

Samples were sequenced in different runs and pooled with other libraries of similar insert sizes. The number of reads obtained per sample reflects and respects their quantities, *i.e.* nmols that were loaded on the sequencer.

***In terms of bioinformatics analysis, it will be required to put the parameters used for each program, in case someone wants to reproduce this. For Kraken2, it is important to know what is the kmer size to index the database. For MEGAHIT assembly it will be important to know the kmer and step sizes used.***

All these details will be provided in the version 2 of the paper.

***For the completeness assessment, the authors used BUSCO, but apparently they are using the whole assembly to assess the completeness. This is not correct, since they must first separate in bins which genomes they have really reconstructed and then they can assess the completeness of them. Probably they can report the an average completeness value for all the reconstructed genomes. By doing the binning they can have a better analysis of what was really reconstructed and how complete it was.***

This is a good point. While our aim was to estimate the total proportion of BUSCO genes that were reconstructed, irrespective of the species of the organism to which they belong, we understand that a practical application is likely to require separating the reconstructed genomes. We will integrate our analysis by binning the reconstructed genomes.

***The use of Krona in Figure 2 is not very convenient. The whole point of a Krona graph is that is interactive. If authors want to provide the Krona data to be downloaded it would be possible and recommended. Having said that, I recommend to use bar plots to represent the relative abundance and composition of the samples at a given taxa level.***

We will either provide a link to interactive krona graphs and/or bar plots reporting the relative abundance and composition of the samples.
